# Circulating Tumor Cells and Metabolic Parameters in NSCLC Patients Treated with Checkpoint Inhibitors

**DOI:** 10.3390/cancers12020487

**Published:** 2020-02-19

**Authors:** Angelo Castello, Francesco Giuseppe Carbone, Sabrina Rossi, Simona Monterisi, Davide Federico, Luca Toschi, Egesta Lopci

**Affiliations:** 1 Nuclear Medicine, Humanitas Clinical and Research Center-IRCCS, 20089 Rozzano, Italy; angelo.castello@cancercenter.humanitas.it; 2 Anatomy and Histopathology, Santa Chiara Hospital, 38122 Trento, Italy; francesco.gcarbone@gmail.com; 3 Oncology and Hematology, Humanitas Clinical and Research Center-IRCCS, 20089 Rozzano, Italy; sabrina.rossi@cancercenter.humanitas.it (S.R.); luca.toschi@cancercenter.humanitas.it (L.T.); 4 Immunology and Inflammation, Humanitas Clinical and Research Center-IRCCS, 20089 Rozzano, Italy; simonterisi@gmail.com; 5 Pathology, Humanitas Clinical and Research Center-IRCCS, 20089 Rozzano, Italy; davide.federico@humanitas.it

**Keywords:** non-small-cell lung cancer, circulating tumor cells, PET/CT, immunotherapy, response to treatment

## Abstract

Circulating tumor cells (CTC) count and characterization have been associated with poor prognosis in recent studies. Our aim was to examine CTC count and its association with metabolic parameters and clinical outcomes in non-small cell lung carcinoma (NSCLC) patients treated with immune checkpoint inhibitors (ICI). For this prospective study, data from 35 patients (23 males, 12 females) were collected and analyzed. All patients underwent an 18F-fluorodeoxyglucose positron emission tomography/computed tomography (18F-FDG-PET/CT) scan and CTC detection through Isolation by Size of Tumor/Trophoblastic Cells (ISET) from peripheral blood samples obtained at baseline and 8 weeks after ICI initiation. Association of CTC count with clinical and metabolic characteristics was studied. Progression-free survival (PFS) and overall survival (OS) were analyzed using the Kaplan–Meier method and the log-rank test. Median follow-up was 13.2 months (range of 4.9–21.6). CTC were identified in 16 out of 35 patients (45.7%) at baseline and 10 out of 24 patients at 8 weeks (41.7%). Mean CTC numbers before and after 8 weeks were 15 ± 28 and 11 ± 19, respectively. Prior to ICI, the mean CTC number was significantly higher in treatment-naïve patients (34 ± 39 vs. 9 ± 21, *p* = 0.004). CTC count variation (ΔCTC) was significantly associated with tumor metabolic response set by European Organization for Research and Treatment of Cancer (EORTC) criteria (*p* = 0.033). At the first restaging, patients with a high tumor burden, that is, metabolic tumor volume (MTV) and total lesion glycolysis (TLG), had a higher CTC count (*p* = 0.009). The combination of mean CTC and median MTV at 8 weeks was associated with PFS (*p* < 0.001) and OS (*p* = 0.024). Multivariate analysis identified CTC count at 8 weeks as an independent predictor for PFS and OS, whereas ΔMTV and maximum standardized uptake value variation (ΔSUVmax) was predictive for PFS and OS, respectively. Our study confirmed that CTC number is modulated by previous treatments and correlates with metabolic response during ICI. Moreover, elevated CTC count, along with metabolic parameters, were found to be prognostic factors for PFS and OS.

## 1. Introduction

The introduction of antibodies against programmed cell death protein-1 (PD-1) and its ligand (PD-L1), a crucial axis involved in the immune surveillance, has prompted encouraging results in the treatment of advanced non-small cell lung carcinoma (NSCLC), although only a minority of patients show clinical response [1]. As a consequence, there is a compelling need to understand the molecular basis of cancer growth and identify potential biomarkers of response, in order to better select patients who will benefit from such new agents.

In the last years, circulating tumor cells’ (CTC) count and characterization have become of great interest in the scientific community [2,3]. Some studies have demonstrated that CTC enumeration is related to poor prognosis in different metastatic malignancies, including lung, breast, colorectal, prostate, and gastric cancer. Furthermore, molecular characterization of CTC might expand our knowledge on tumor heterogeneity, especially in patients for whom tissue biopsies are difficult to perform [4,5,6,7,8].

Molecular imaging, using 18F-fluorodeoxyglucose (18F-FDG) with positron emission tomography/computed tomography (PET/CT), is widely applied in oncology as a useful marker of tumor biology. Indeed, by differentiating higher versus less-active metabolic tumor tissues, semi-quantitative metabolic parameters can offer the possibility for non-invasive, in vivo tumor characterization and for correct evaluation of tumor response [9,10,11]. However, available studies analyzing the association between 18F-FDG PET/CT and CTC in NSCLC are limited to chemotherapy-naïve patients or those treated with “traditional” antitumor drugs [12,13,14,15].

On the basis of these premises, our aim was to examine CTC count in NSCLC patients treated with immune checkpoint inhibitors (ICI) and determine its relationship with metabolic parameters by 18F-FDG PET/CT and clinical outcomes.

## 2. Results

### 2.1. Patients’ Characteristics

A total of 20 patients (57.1%) received nivolumab, 12 (34.3%) pembrolizumab, 2 patients (5.7%) had a combination of nivolumab and ipilimumab, and only 1 (2.9%) patient was treated with atezolizumab. The median number of immunotherapy cycles was 8 (range of 1–47). Median follow-up was 13.2 months (range of 4.9–21.6 months).

### 2.2. CTC and Clinic-Pathologic Features

CTC were identified (CTC ≥ 1) in 16 out of 35 patients (45.7%) at baseline prior to ICI therapy, and the CTC count ranged between 0 and 130 (mean ± standard deviation (SD), 15 ± 28). The minimum number of CTC detected was 5, in particular 8 patients had a CTC count between 5 and 20, whereas the other 8 patients had CTC from 25 to 130.

At the first restaging, peripheral blood samples were available for 24 patients because of progression of disease or a worsening of clinical conditions in the other cases. CTC were detected in 10 out of 24 patients (41.7%). The median number of CTC was 11 ± 19 in 10 mL of blood. CTC count ranged between 5 and 20 in six patients, and between 30 and 60 in the other four patients. In addition, we demonstrated a reduction of CTC in nine patients (37.5%), unchanged in eight patients (33.3%), and increased in seven patients (29.2%). Although a reduction in the mean number of CTC before and after 8 weeks of treatment was detected, this decrease was not statistically significant.

**Figure 1:**
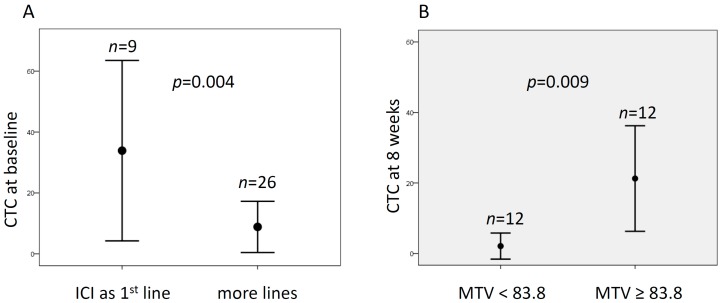
Association of circulating tumor cells (CTC) count with clinical-metabolic features. (**A**) Mean number of CTC at baseline according to the number of previous lines of treatment. (**B**) Mean number of CTC at the first restaging (about 8 weeks) and the median value of metabolic tumor volume (MTV).

The association between CTC count and patient characteristics was explored, both before treatment and at the first restaging. There was a statistically significant association between CTC count and previous treatments. Of note, patients who underwent ICI as first-line treatment had a mean number of CTC at baseline higher than patients who started ICI after more lines of treatment (34 ± 39 vs. 9 ± 21, *p* = 0.004) (Figure 1A). Likewise, a trend was observed with high baseline CTC count and pembrolizumab (*p* = 0.09); indeed, the latter is often used in first-line settings. No further association was found between CTC counts, as well as the other clinical variables, such as age, gender, smoking history, and tumor type.

### 2.3. Relationship of CTC and Tumor Response

Of the 35 patients enrolled, 31 patients underwent tumor assessment by computed tomography (CT), whereas 18F-FDG PET/CT scans were available from 28 patients at the first response assessment. According to Response Evaluation Criteria In Solid Tumors (RECIST) 1.1, partial response (PR) was observed in 6 patients, stable disease (SD) was observed in 12, and progressive disease (PD) in 13. We found that patients with PD showed a trend toward higher baseline CTC count (26 ± 36) compared with patients with partial response (14 ± 14) or stable disease (9 ± 26) (*p* = 0.076). There was no significant difference in the CTC count after 8 weeks among the three groups of response.

According to the European Organization for Research and Treatment of Cancer (EORTC) criteria, partial metabolic response (PMR), stable metabolic disease (SMD), and progressive metabolic disease (PMD) were observed in 10, 6, and 12 patients, respectively. There was no significant difference in the baseline CTC count, as well as after 8 weeks, among the three groups. However, considering CTC changes (ΔCTC) within individual patients, among the 24 patients that had their CTC analyzed after 8 weeks of treatment, we found that the increase of CTC count was associated with poor response to ICI by means 18F-FDG PET/CT, as PMD rates were significantly different between patients with CTC increase at 8 weeks and patients with stable or decreased number of CTC (71.4% vs. 28.6% vs. 0%, respectively, *p* = 0.033) (Table 1).

### 2.4. CTC and Semi-Quantitative 18F-FDG Parameters

The median maximum standardized uptake value (SUVmax), average SUV (SUVmean), metabolic tumor volume (MTV), and total lesion glycolysis (TLG) before the initiation of treatment were 13.5 (range of 4.9–35.7), 5.9 (3.2–9.8), 68 (8–1772), and 362.8 (31–2504), respectively. Median SUVmax, SUV mean, MTV, and TLG after 8 weeks were 12.1 (3.6–38.4), 5.6 (3–13.8), 83.8 (2.5–623.3), and 511.2 (7.6–4332.7), respectively. Median ∆SUVmax, ∆SUVmean, ∆TLG, and ∆MTV were −12.9% (−75.5–107.1%), −0.88% (−61.3–130%), 47.8% (−99.4–1295%), and 30.4% (−98–1245%). At baseline, the number of CTC did not correlate with metabolic parameters, and only a trend for SUVmax was observed (*p* = 0.072). Conversely, after 8 weeks of treatment, CTC count was significantly associated with metabolic volume, expressed by MTV and TLG. Indeed, patients with MTV and TLG above the median values had higher mean number of CTC than patients with low metabolic tumor burden (both MTV and TLG *p* = 0.009) (Figure 1B). No difference was found between CTC and percentage changes of metabolic parameters.

### 2.5. Relationship between CTC Count, 18F-FDG PET Parameters, and Survival

**Figure 2:**
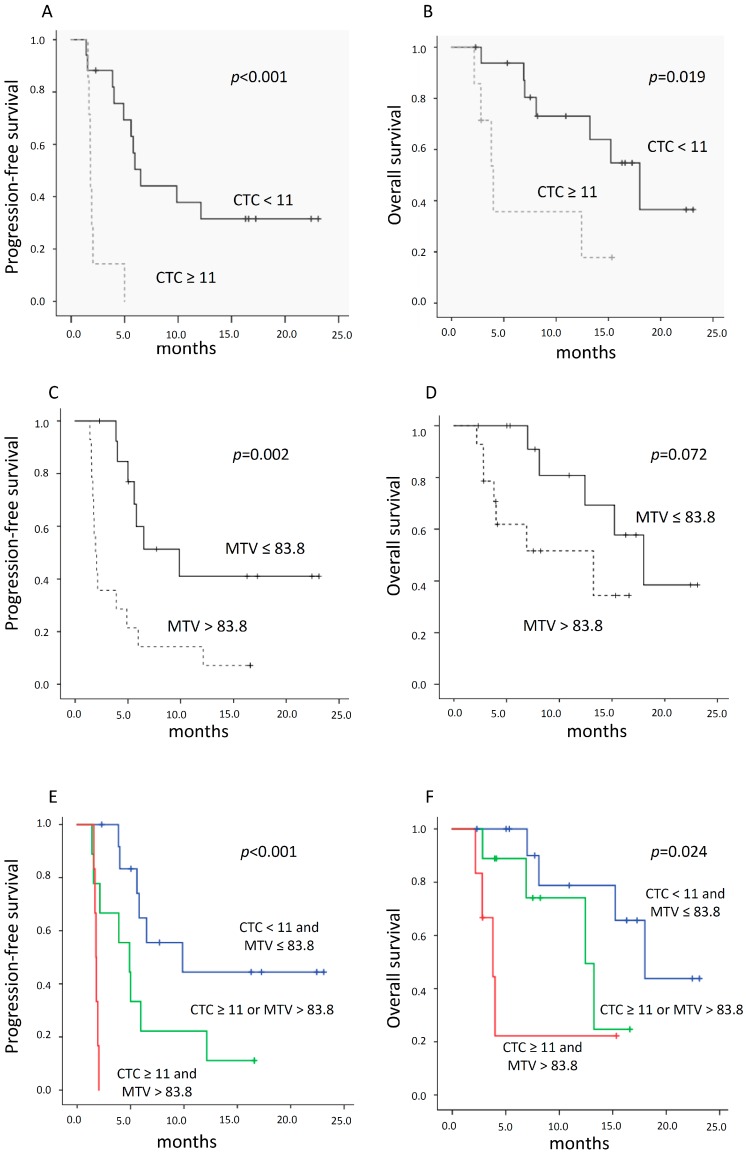
Kaplan–Meier curves according to CTC count and metabolic parameters. (**A**,**B**) Progression-free survival (PFS) and overall survival (OS) according to CTC count at 8 weeks, below or above the mean value. (**C**,**D**) PFS and OS of patients with MTV at 8 weeks greater or lower than median value. (**E**,**F**) PFS and OS according to the combination of mean number of CTC and median MTV after 8 weeks of treatment.

The median progression-free survival (PFS) and overall survival (OS) of patients with CTC counts ≤ 11 after 8 weeks were 6.5 months (range of 5.1–7.9 months) and 18 months (range of 12–24 months), respectively. The median PFS and OS of patients with CTC counts > 11 were 1.8 months (range of 1.7–1.8 months) and 4 months (range of 2.7–5.3 months), respectively. The differences in PFS and OS were both statistically significant (*p* < 0.001, *p* = 0.019 for PFS and OS, respectively) (Figure 2A,B).

**Figure 3:**
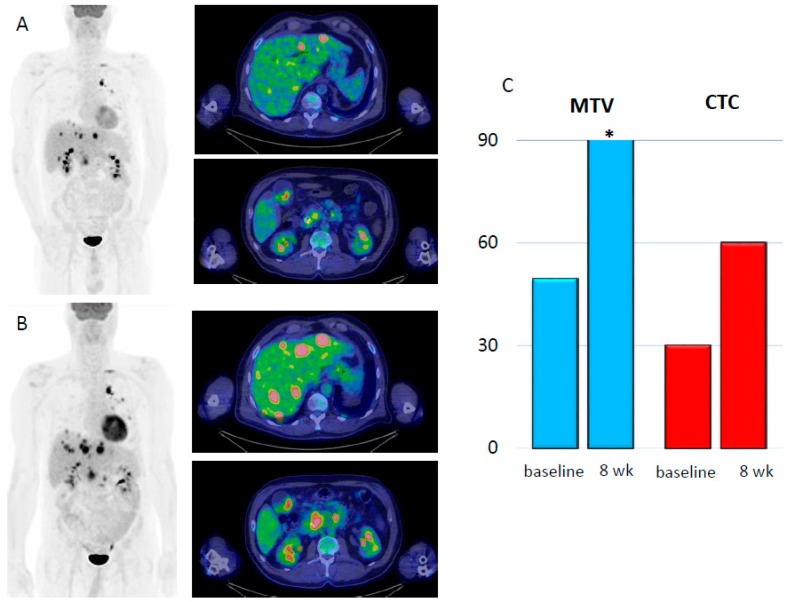

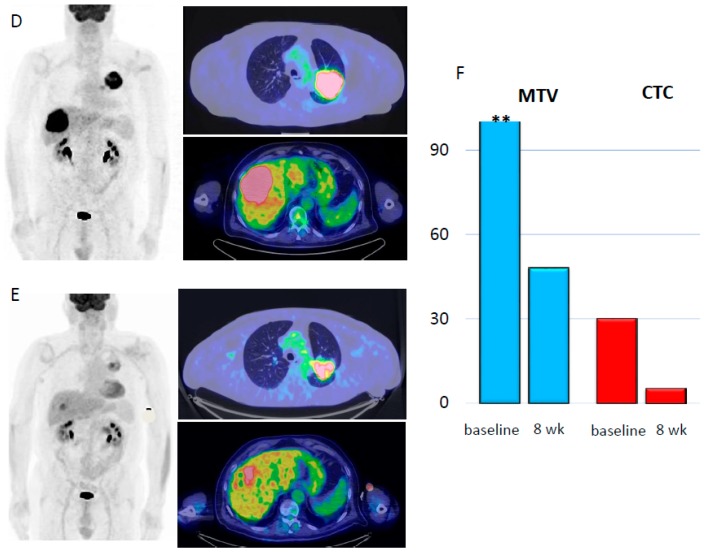
Two cases of progression (**A**–**C**) and response (**D**–**F**) to ICI according to metabolic parameters and CTC evaluation. (**A**) Maximum intensity projection (MIP) with two axial slices of liver and celiac node metastases at baseline. (**B**) Increase of tumor burden and appearance of further metastatic sites within the liver and in the abdominal nodes at the first restaging. (**C**) Bar graph representation of MTV (blue bars) between the baseline (49.4 mL) and the first restaging (97.2 mL). Likewise, CTC count (red bars) increased from 30 to 60. (**C**) MIP with two large lesions within the lung and liver at baseline. (**D**) 18F-fluorodeoxyglucose positron emission tomography/computed tomography (18F-FDG PET/CT) at 8 weeks, which demonstrated a decrease of overall tumor burden. (**F**) Bar graph representation of MTV (blue bars) at baseline (256.5 mL) and at 8 weeks (48 mL). Likewise CTC count (red bars) decreased from 30 to 5, * MTV= 97.2 mL; ** MTV= 256.5 mL.

The median PFS and OS of patients with median MTV ≤ 83.8 were 9.9 months (range of 3.6–16.1 months) and 18 months (range of 12.6–23.4 months), respectively. The median PFS and OS of patients with median MTV > 83.8 were 1.9 months (range of 1.5–2.4 months) and 13.2 months (range of 2–24.5 months), respectively. The difference for PFS was statistically significant (*p* = 0.002), whereas for OS it showed only a trend (*p* = 0.072) (Figure 2C,D). Furthermore, we tested whether the combination of CTC count and MTV at 8 weeks could provide further discriminatory value in predicting clinical outcomes. Of note, all patients with MTV ≤ 83.8 and CTC ≤ 11 had the longest PFS and OS. Among patients with median MTV greater than 83.8, those with CTC count ≤ 11 were associated with longer PFS and OS than patients whose CTC were above the mean value (Figure 3E,F) (*p* < 0.001 and *p* = 0.024 for PFS and OS, respectively). Regarding percentage changes of metabolic parameters, we found that ΔMTV and ΔTLG were associated with PFS (both 9.9 vs. 2.1 months, *p* = 0.010 and *p* = 0.009, respectively), whereas ΔSUVmax was prognostic for OS (median not reached vs. 12.4 months, *p* = 0.013).

Finally, due to the low number of events, only three parameters were included in the multivariate Cox analysis. Of note, the number of CTC at the first restaging was confirmed as a predictive factor for PFS and OS, along with ΔMTV for PFS and ΔSUVmax for OS (Table 2).

**Figure 4:**
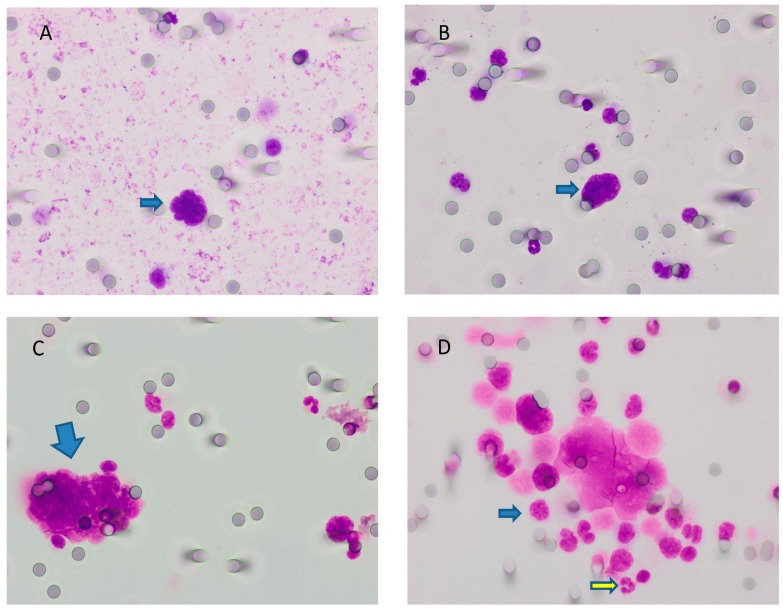
Comparison of positive (**A**–**C**) and negative findings (**D**) for CTC visualized by May–Grünwald–Giemsa (MGG) staining at 40× magnification. (**A**) Naked nucleus (without cytoplasm), intensely stained (hypercromatic), with irregular shape and scalloped borders, suspect for carcinoma. The absence of a clearly visible cytoplasm did not permit the evaluation of the nuclear/cytoplasmic ratio. (**B**) The same as the previous image; herein, we can compare the size of the naked nucleus (arrow) with the size of neighboring leukocytes (around 3–4 times bigger). (**C**) A cluster of cells with a high nucleus/cytoplasm ratio, hypercromasia, and irregular shape of nuclei. Additionally, cells were bigger than leukocytes. (**D**) Some of the cells in this sample were degenerated. We did not take into account these cells. The remaining population (for example, the cell indicated by the blue arrow) had a nuclear size somewhat similar to that of leukocytes (yellow arrow). Where cytoplasm was present, it was granular. Thus, all of these cells were probably leukocytes. * CTC = circulating tumor cells; MGG = May–Grünwald–Giemsa.

Figure 4A,B shows two cases with metabolic response by 18F-FDG PET/CT and CTC assessment before and after 8 weeks of ICI.

## 3. Discussion

Several studies have investigated the prevalence and the prognostic role of CTC in different cancer types, including NSCLC, but only a limited number have analyzed their relationship to 18F-FDG PET/CT parameters [13,16,17,18]. On the other side, only a few studies have assessed the role of CTC in patients with advanced NSCLC treated with checkpoint inhibitors [19,20]. If we exclude our preliminary data [21], to the best of our knowledge, this research is the first to report a significant association between the CTC count and the tumor 18F-FDG uptake in a similar patient cohort treated with immunotherapy.

In our analysis, CTC were detected in 46% of the patients before the initiation of ICI and in 42% at the first assessment. Our detection rate was superior compared to Tamminga’ study [20], that being 32% before ICI and 27% after 4 weeks, where CTC identification was performed by epithelial marker-dependent (CellSearch) technology. Moreover, our finding is consistent with previous studies in lung cancer patients, which demonstrated an overall sensitivity higher for cell size rather than marked-based approaches, although not in an ICI setting [22,23,24,25,26]. Nevertheless, in the abovementioned study from the Italian group [19], using another system based on cell size (i.e., Screencells Cyto), the prevalence of CTC was almost double that of our study (91% vs. 46%) in a larger population (*n* = 89). This discrepancy by the two techniques, although based on the same filter diameter, might suggest a high inter-reader variability due to both readers’ skills and the lack of uniformly accepted criteria for CTC definition. Therefore, further and larger studies are needed.

Similar to Krebs et al., in our study, the presence and the number of CTC were influenced by previous lines of therapy. Indeed, patients with a positive history for previous therapy had a mean number of CTC lower than those who underwent ICI as first-line therapy (*p* = 0.004) [7]. As a consequence, the presence of CTC in the peripheral blood after chemotherapy might suggest the grade of response to treatment or, in other words, the aggressiveness of the tumor determining how fast cancer can return after a macroscopic response. Hence, residual CTC after chemotherapy could be characterized in order to identify those morphological or genetic modification-inducing expression of genes and proteins conferring drug-resistance, such as the endothelial to mesenchymal transition observed in cancer stem cells [27,28]. In line with previous studies, we did not find a significant association between CTC and clinicopathological characteristics (e.g., age, gender, tobacco exposure, tumor size, and histologic subtype) in patients with advanced NSCLC [14,29,30]. Moreover, CTC count both at baseline and after 8 weeks, as well as their change during treatment, was not associated with tumor response according to morphologic criteria (RECIST 1.1), although a trend of significance with higher mean number of CTC for the PD group (*p* = 0.076) was evident. Such a finding was consistent with the two abovementioned studies in NSCLC patients treated with ICI [19,20]. Of note, CTC were associated with durable response, defined as no progression for at least 6 months measured by RECIST 1.1, more pronounced than early tumor response at 4–6 weeks [20]. Likewise, Nair et al., prior to any therapeutic intervention for NSCLC, reported no correlation for CTC and tumor diameter [13]. On the other hand, considering EORTC criteria, we showed a significant association between patients with increased CTC and poor metabolic response by 18F-FDG PET/CT at first assessment after 8 weeks of treatment. Similarly, Punnoose and colleagues [15] demonstrated higher levels of CTC in patients classified as non-responders by metabolic criteria, although this was performed in a cohort treated with erlotinib and pertuzumab. Hence, our results confirm on one hand the prevailing cytostatic effect of ICI compared to cytocidal and, on the other, the precocious metabolic changes detected by 18F-FDG that occur earlier than morphologic changes.

We also investigated the relationship between CTC and metabolic 18F-FDG positron emission tomography (PET)-based indexes. Interestingly, we detected a significant association between higher densities of CTC after 8 weeks and metabolic tumor burden, expressed by MTV and TLG (both *p* = 0.009). A significant difference was also found between the number of CTC at 8 weeks and the percentage changes of metabolic volume (i.e., ∆MTV and ∆TLG). These findings suggest, as already stated previously, that the CTC count in the peripheral blood can reflect the entity of the tumor burden and provide valuable information on the metabolic activity, which may serve as a marker of tumor aggressiveness in advanced NSCLC. Hence, as CTC count is a marker of poor response, if our results were confirmed in a larger cohort, CTC along with metabolic indexes would be useful for monitoring disease, allowing for early cessation of treatment with checkpoint inhibitors, and switching to an alternative therapeutic regimen. Because our paper is the first in the era of checkpoint inhibitors investigating CTC and 18F-FDG PET parameters, comparison with other reports is not well applicable. Previously, some studies demonstrated a significant correlation between CTC and SUV value in patients with chemotherapy-naïve lung cancer [12,13,14,31]. On the contrary, Nygaard et al. did not find any association between metabolic parameters and cell-free (cf) DNA, another tumor-derived biomarker [32].

The presence of CTC is related with survival and is predictive of disease progression and death in NSCLC during chemotherapy and targeted therapies [15,23,29,33,34,35,36,37]. In the present study, CTC count after 8 weeks was significantly associated with PFS and OS, whereas MTV at first restaging was prognostic only for PFS, and showed a trend for OS (*p* = 0.072), in our opinion due to limited sample size. In addition, CTC and MTV were also prognostic factors when considered in conjunction. In this regard, we identified a group characterized by poor PFS presenting with high CTC and high MTV at 8 weeks. Our findings are consistent with the only two abovementioned studies, which explored the prognostic role of CTC in patients receiving ICI [19,20]. Interestingly, Tamminga et al. showed that CTC and cell-free DNA (cfDNA), although at baseline, separately and in conjunction, were significantly associated with OS in NSCLC patients receiving nivolumab [20]. Moreover, CTC count after 8 weeks was predictive for both PFS and OS, along with ΔMTV and ΔSUVmax for PFS and OS, respectively, suggesting that large and highly metabolic tumors could have the potential of shedding a high number of CTC in the bloodstream, increasing the possibility to metastasize at distant sites.

These findings may be particularly interesting for patients in whom no tumor tissue is available for other predictive analysis. On the same line, Fiorelli et al. identified SUVmax as an independent predictor for CTC presence after surgery in NSCLC patients [31].

Nevertheless, our study had some shortcomings. First, a limited sample size, and second, we did not evaluate PD-L1 expression on CTC. Recently, Ilié et al. demonstrated that patients who had PD-L1-negative CTC 6 months after the start of checkpoint inhibitors benefitted from immunotherapy, highlighting CTC as a heterogeneous population worthy of further investigation [33]. Third, we did not investigate the genetic features of CTC, which could open a new frontier in the understanding of therapy resistance. Finally, we did not collect other circulating markers, such as cfDNA, known as potential biomarkers in cancer patients. 

## 4. Materials and Methods

### 4.1. Patients and Study Design

The current study was conducted following the approval of the local institutional review board and in accordance with the Declaration of Helsinki and Good Clinical Practice guidelines (Prot. Nr. CE Humanitas ex D.M. 8/2/2013 335/17). Written informed consent was obtained in all cases. The trial was registered at www.clinicaltrials.gov (NCT03563482). Between April 2017 and March 2019, 35 patients (23 males, 12 females) affected by metastatic or relapsed NSCLC were referred to our hospital, Humanitas Clinical and Research Center, for treatment with ICI, and were prospectively enrolled. ICI therapy was administered intravenously at a dose of 3 mg/kg every 2 weeks for nivolumab or at a fixed dose of 200 mg every 3 weeks for pembrolizumab.

All patients performed whole-body contrast-enhanced CT, 18F-FDG PET/CT scan, and peripheral blood sample for CTC isolation at baseline and at the first restaging after approximately 8 weeks (after three cycles for pembrolizumab and atezolizumab, and after four cycles for nivolumab). The patients’ epidemiologic and clinical characteristics are reported in Table 3.

### 4.2. CTC Isolation and Enumeration

For CTC detection, 10 mL of blood was collected in EDTA (ethylenediaminetetraacetic acid) tubes and processed within 2 h on the Isolation by Size of Tumor/Trophoblastic Cells (ISET) platform (Rarecells, Paris, France). Peripheral blood was filtered through the ISET polycarbonate membrane containing 10 filter-spots with calibrated 8 μm diameter cylindrical pores, each spot representing the filtration of 1 mL of blood. The membrane was cut into two parts containing four and six spots per part. Four spots were stained using a freshly made May–Grünwald–Giemsa (MGG) solution according to the technique described by Hofman and colleagues [29] for 5 min with undiluted May–Grünwald, and subsequently for 5 min with 50% diluted May–Grünwald and 40 min in 10% diluted Giemsa, followed by rinsing with water. Membranes were then air-dried and mounted with limonene mounting medium (Sigma-Aldrich, St. Louis, MO, USA) and kept in the dark at room temperature. Stained spots were examined under a light microscopy (Olympus BX51, Olympus Corporation, Shinjuku, Tokyo, Japan) at 10× and subsequently digitized at 40× magnification. All images were analyzed by two cytopathologists blinded to the study data. CTC were recognized on the basis of four cytopathological features: (a) nuclear hyperchromatism, (b) increased nuclear volume, (c) irregular nuclear borders, and (d) increased nucleus-to-cytoplasm ratio. Cells were defined as CTC when all four abovementioned criteria were fulfilled, as previously described by Hofman and colleagues [29]. A dedicated pathologist analyzed and counted CTC present in the membranes (four spots per patient). A panel of features obtainable after magnification is illustrated in Figure 3.

### 4.3. 18F-FDG PET/CT and Image Analysis 

Patients fasted at least 6 h before intravenous administration of 250–500 MBq of fluorodeoxyglucose (FDG) in a quiet room. Images were acquired 60 min after tracer injection using two scanners accredited by EANM Research Ltd. (EARL) program [38]: (a) Siemens Biograph LSO (lutetium oxyorthosilicate) 6 scanner (Siemens Erlangen, Munich, Germany), with an integrated 6-slice CT; and (b) GE Discovery PET/CT 690 (General Electric Healthcare, Waukesha, WI, USA), with an integrated 64-slice CT. Attenuation-correction images were obtained with a low-dose CT (120 kV, 30 mA). Unenhanced low-dose CT was performed at 140 kV and 40 mA for attenuation correction of emission data and anatomic localization of the PET dataset. PET sinograms were reconstructed by means of an ordered-subset expectation maximization iterative reconstruction algorithm (three iterations; eight subsets). Images were displayed on a GE ADW4.6 workstation (GE Healthcare, Waukesha, WI, USA) and interpreted by two experienced nuclear medicine physicians. From 18F-FDG PET/CT images, the following parameters were measured: SUVmax, SUVmean, MTV, and TLG; MTV was assessed using a PETVCAR (GE Healthcare, Waukesha, WI, USA) workstation and was computed using an SUVmax threshold of 41%; TLG was computed as MTV × SUVmean. Percentage reduction between baseline and restaging was calculated using the formula: [(8 weeks SUVmax − pretreatment SUVmax)/pretreatment SUVmax] × 100 for SUVmax; similarly for the other metabolic parameters.

### 4.4. Tumor Response Assessment

Early tumor response, after approximately 8 weeks of treatment, was measured using the revised RECIST 1.1 criteria [39]. Four categories were identified: complete response (CR), disappearance of all target lesions; PR, reduction of at least 30% in the sum of diameters of target lesions; PD, increase of at least 20% in the sum of diameters of target lesions or appearance of new lesions; SD, neither CR, nor PR or PD.

Metabolic response was evaluated according to EORTC criteria with the following categories: complete metabolic response (CMR), complete resolution of 18F-FDG uptake within all lesions; PMR, reduction of at least 25% in the sum of SUVmax; PMD, increase of at least 25% in the sum of SUVmax or appearance of new 18F-FDG avid lesions that are typical of cancer and not related to inflammation or infection; SMD, neither CMR, nor PMR or PMD [40].

### 4.5. Statistical Analysis 

Descriptive statistics for clinical, imaging, and pathologic variables were determined using the median (range) or media with SD as appropriate.

Associations of CTC with clinical and metabolic characteristics were studied by means of *t*-tests and Mann–Whitney U tests for continuous variables and χ^2^ tests or Fisher’s exact test for categorical variables.

Clinical outcomes were evaluated in terms of PFS, defined as the interval from the date of initiation of ICI to the date of either disease progression or death, and OS calculated as the duration between the date of initiation of immunotherapy and the date of death. For the univariate and multivariate analyses of survival, Cox’s proportional hazard model was employed as well as the log-rank test with Kaplan–Meier analysis. All statistical analyses were carried out using the Statistical Package for Social Sciences, version 23.0, for Windows (SPSS, Chicago, IL, USA), and *p*-values < 0.05 were considered as being statistically significant.

## 5. Conclusions

In our study, analyzing for the first-time concomitant CTC and metabolic parameters in NSCLC patients receiving ICI, we observed that CTC number was modulated by previous therapeutic interventions. Moreover, the presence of elevated CTC count was an additional prognostic and predictor factor along with metabolic tumor burden. Further large-scaled studies confirming the clinical utility of CTC in combination with metabolic PET-based parameters in this setting are now warranted.

## Figures and Tables

**Table 1:** Association between circulating tumor cells (CTC) count and response to immune checkpoint inhibitors (ICI).

Parameter	EORTC		
	CMR/PMR	SMD	PMD
Baseline CTC count			
CTC ≤ 15 (*n* = 18)	27.8% (5)	33.3% (6)	38.9% (7)
CTC > 15 (*n* = 10)	50% (5)	0% (0)	50% (5)
*p*-value	ns	ns	ns
ΔCTC count after 8 weeks			
decreased (*n* = 9)	66.7% (6)	11.1% (1)	22.2% (2)
stable (*n* = 8)	12.5% (1)	37.5% (3)	50% (4)
increased (*n* = 7)	0% (0)	28.6% (2)	71.4% (5)
*p*-value	ns	ns	0.033

CMR/PMR, complete metabolic response/partial metabolic response; SMD, stable metabolic disease; PMD, progressive metabolic disease. ns: not significant.

**Table 2:** Uni- and multivariate Cox proportional hazard regression analyses for the prediction of PFS and OS.

Parameters	PFS			OS		
	Hazard Ratio	95% IC	*p*-Value	Hazard Ratio	95% IC	*p*-Value
Age (median)	1.233	0.564–2.694	ns	1.009	0.423–2.910	ns
Gender	0.346	0.156–0.768	0.009	0.329	0.119–0.905	0.031
Smoking history	1.407	0.480–4.129	ns	3.518	1.050–11.790	0.041
Histology	0.828	0.345–1.990	ns	0.907	0.291–2.822	ns
PD-L1 status	0.736	0.255–2.128	ns	0.659	0.147–2.957	ns
CTC baseline (median)	1.089	0.472–2.512	ns	1.869	0.607–5.759	ns
CTC at 8 weeks (median)	0.135	0.040–0.458	0.001	0.260	0.77–0.871	0.029
SUVmax baseline (median)	1.049	0.483–2.276	ns	0.996	0.383–2.654	ns
SUVmean baseline (median)	0.701	0.320–1.534	ns	0.839	0.312–2.257	ns
TLG baseline (median)	0.998	0.459–2.171	ns	1.016	0.389–1.534	ns
MTV baseline (median)	1.601	0.739–3.468	ns	2.473	0.934–6.551	ns
∆SUVmax (median)	2.409	0.954–6.085	ns	0.179	0.039–0.825	0.027
∆SUVmean (median)	1.498	0.611–3.671	ns	0.375	0.100–1.402	ns
∆TLG (median)	0.310	0.122–787	0.014	0.751	0.241–2.346	ns
∆MTV (median)	0.312	0.123–792	0.014	0.481	0.151–1.532	ns
Multivariate Cox proportional hazards regression analysis						
CTC at 8 weeks (median)	0.115	0.030–0.434	0.001 *	0.178	0.045–0.707	0.014 *
ΔMTV (median)	0.357	0.130–0.984	0.046 *			
ΔSUVmax (median)				0.144	0.028–0.736	0.02 *

* *p* < 0.05.

**Table 3:** Patients’ characteristics at baseline.

Characteristics	*N* (%)
**Patients**	35
**Median age (range)**	77 (51–86)
**Gender**	
Male	23 (65.7)
Female	12 (34.3)
**Smoking status**	
Never	5 (14.3)
Former	19 (54.3)
Smoker	11 (31.4)
**ECOG PS**	
0	18 (51.4)
≥1	17 (48.6)
**Therapy line**	
0	9 (25.7)
1	14 (40)
≥2	12 (34.3)
**Metastatic sites**	
1–2	16 (45.7)
>2	19 (54.3)
**Histology**	
Adeno	24 (68.6)
Squamous	7 (20)
Poorly differentiated	34 (8.6)
Sarcomatoid	1 (2.8)
**Tumor PD-L1 status**	
Negative	7 (20)
Positive	14 (40)
Not evaluable *	14 (40)

* Programmed cell death ligand 1 (PD-L1) could not be evaluated in 14 patients as biopsied material was of insufficient quality or quantity.
